# Traumatic Separation of the Epiphyses

**Published:** 1899-09

**Authors:** 


**Traumatic Separation of the Epiphyses.** By John Poland. Pp. xxxi., 926. London: Smith, Elder, & Co. 1898.

This is a most exhaustive treatise, and is the result of investigations by the author, which have been carried on for many years, both in clinical and in anatomical work. We could not have imagined that so large a book could have been written on the subject. It is one to which all surgeons may from time to time turn for information, if they suspect the injury from which their patient suffers to be of this nature, and they will certainly not be disappointed by the information they will receive. Illustrations are very abundant, and most of them very good, and there can hardly have been a book yet published with so many illustrations of skiagrams. Some of these are very clear, but in a few we must say we fail to see a demonstration of the lesion. We would suggest to the author that a series of skiagrams of the normal epiphyses of all the bones of the extremities would be a very valuable addition to the next edition of the book, for in skiagraphic work the picture of a normal part is of very great assistance. Nineteen skiagrams of the normal epiphyses of the hand and wrist at various ages are given, and one or two of each of the long bones would be a great gain. The author first considers traumatic separation of the epiphyses in general, and then describes in a most thorough way the pathology, signs, and treatment of those lesions in the various bones. He has collected an enormous number of cases to illustrate the subject.

The book is issued in a state of magnificence that has rarely been equalled in medical work, and we feel sure that it will be the standard English work on the subject; our knowledge regarding it has been very greatly increased by the author.

